# Role of Sociocultural Factors in Depression among Elderly of Twin Cities (Rawalpindi and Islamabad) of Pakistan

**DOI:** 10.1155/2014/230737

**Published:** 2014-09-21

**Authors:** Saira Javed

**Affiliations:** Department of Behavioral Sciences, Fatima Jinnah Women University, The Mall, Rawalpindi 46000, Pakistan

## Abstract

This research was conducted to examine the role of sociocultural factors on depression among elderly of twin cities (Rawalpindi and Islamabad) of Pakistan. 310 older adults participated in the present study. Through convenient sampling technique, face to face interview was carried out for data collection. Urdu translated Geriatric Depression Scale Short Form and demographic sheet were used to test hypotheses. Descriptive statistics and *t*-test were used for data analysis. Results showed significant mean differences among gender, marital status, family system, and status of employment on depression. Financial crisis, feeling of dejection because of isolation, and trend of nuclear family system have been observed as strong predictors of depression in older adults.

## 1. Introduction/Literature Review

Depression enduring geriatrics was associated with increased medical morbidity, impaired body working, disturbed social functioning, and dementia [1, 2]. Depression is illustrated by insufficient sleeping hours, low or absence of diet, mood swings, glumness or isolation, and suicide ideation [3]. Pathology of depression in older adults has been widely discussed and assessed by researchers in present era. Researchers narrated that comparative to the other age groups elderly experienced elevated prevalence of depression in older people [4] In 2006 more than 400 million sufferers of depression were recorded in the world. Frequency in cases of depression horribly amplified and stood on fourth position according to global burden of diseases. Depression was announced an alarming mental health disorder. In developing countries, it is expected to be on second rank by the year 2020 [5].

Studies conducted by Pakistani researchers revealed that depressive elderly are severely ignored by common population because of lack of awareness. Either they went under or over recognition of depression specifically in developing countries like Pakistan [6, 7]. Though, in 2008 almost 30% incidence of depression has been found in local study conducted on elderly [8].

Biological studies quoted number of times about the physical superiority of women but they lack in reporting the emotional instability of women that leads to mental health issues in later life. A series of studies conducted in 2005 and 2006 demonstrated that female older adults are more vulnerable to later life depression as compared to male older adults [3, 9]. In 2003 a published research study reported the incidence of depression in women is thrice higher than men [10].

In later life females experienced internal marital conflicts that are responsible of marital distress [11]. Result of longitudinal study explains risk of having depressive episodes in later life is directly associated to marital conflicts caused by aging factors [12, 13]. Recently an extensive literature review done in 2011 documented that single elderly, either widowed, divorced, or unmarried are on greater risk of having depression as compared to married and with their siblings and spouse [14].

Family system acts significantly in Asian society. It has been seen that elderly belongs to rural families are less depressed than those of urban families [15]. Furthermore, Pakistani researchers examined that the incidence of having depression in older adults living in extensive family system is four times lesser than elderly living in nuclear family system [6].

Like other countries, the common age of retirement in Pakistan is 65 years. After retirement it has been seen that mostly people get relaxed and start doing the things that they missed for so many years. So, according to researchers phase of retirement is not at all depressive stage [11]. Instead they called it honeymoon period. On the other hand elderly who belong to private sector face the black side of later life because of financial issue caused by unemployment [16].

Sociocultural factors consistently have been recognized as chief factor in signifying the unpredictability in the prevalence of depression in elderly. With the advancement in modernization, cases of physical and mental health issues are increasing day by day and society become mechanical [17]. The lust of fame and money makes an individual deprived of sociocultural values. Because of this novel behavior, elderly are significantly affected by health issues including depression. Additionally, stress full life events, ignorance from family members, loss of loved ones, financial crises, phase of retirement, and inter- or intrapersonal conflicts accompanied with other health issues lead to late life depression [11, 18].

### 1.1. Objective

The main objective of the present study is to see the influence of sociocultural factors on occurrence of depression in older adults.

### 1.2. Hypotheses 

The following hypotheses are presented in the paper.

1. There exists significant difference in depression between men and women of older adults.
2. There exists significant difference in depression between married and unmarried older adults.
3. There exists significant difference in depression between older adults living in nuclear and extensive family system.
4. There exists significant difference in depression between employed and unemployed older adults.

## 2. Research Methodology

Data were collected through convenient sampling technique from the twin cities of Pakistan that is, Rawalpindi and Islamabad. Design of the present study was descriptive and quantitative. For data collection face to face survey interview was conducted. 310 elderly were participated in the study by signing written inform consent by significant others on their behalves. Urdu translated 15-item Geriatric Depression Scale (GDS-SF) [6] and demographic profile were used to test hypotheses. 10 to 15 minutes were taken by each individual.

Data of the study was analyzed by using SPSS version 14.0. For elaboration of results tables were used for inclusive view of findings. *P* < 0.05 level of significance was set for each observation. *t*-test was applied to determine the differences on depression. Where 0.76 cronbach's alpha reliability was recorded of the study.

## 3. Results

### 3.1. Characteristics of the Participants

Table 1 of the study shows characteristics of elderly participants of twin cities of Pakistan. Category of unmarried elderly also includes 40 widows and 23 divorced. Likely the category of unemployed contains 42 retired older adults. Most of the geriatrics lie in the category of body complaints experienced muscular pains, digestive problems, and gastric issues.

**Figure 1:**
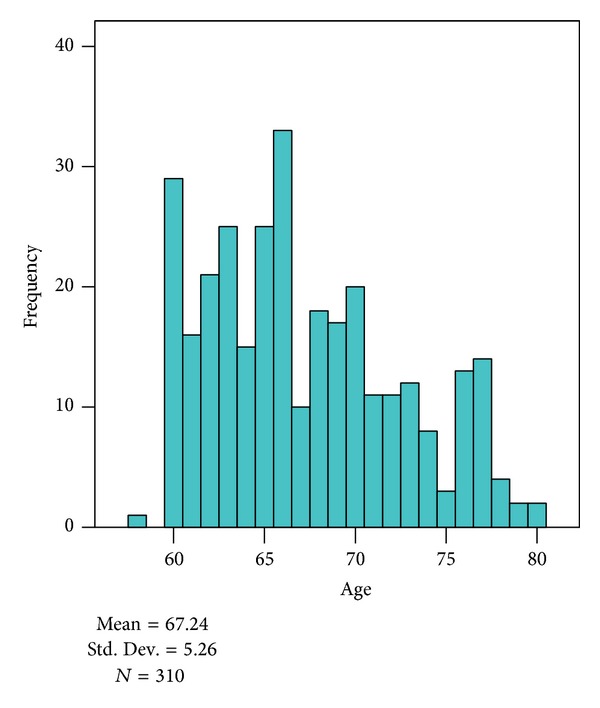
Age distribution of older adults who participated in the study.

Figure 1 shows the age distribution of older adults participated in present study. 67.24 mean age is recorded of 310 elderly, where 189 were from Rawalpindi and 121 were from Islamabad.

### 3.2. Role of Sociocultural Factors in Depression among Elderly

Table 2 indicates the mean differences in various demographic variables among elderly on depression. Results indicate that there are significant mean differences (*P* < 0.05) between men and women, married and unmarried, nuclear family and extensive family system, and employed and unemployed on depression.

## 4. Discussion

Present survey was conducted to investigate about the role of sociocultural factors on depression among elderly of twin cities of Pakistan. Like previous literature, findings of the study revealed significant mean differences in gender, marital status, family system, and status of employment. So, all hypotheses of the study prove to be true (see Table 2).

Asian women in later life were not able to cope up with daily life issues and house chore as she did before because of the old age factor. On the other way, unlike older women of western world, mostly Pakistani women are not working nor found of social gatherings so they more rapidly get isolated that in short time converted in to depression. It has been observed that elder women get depressed sooner as compared to men. Feeling of dejection and fear of death is very much prominent in older adult women as compare to men [19].

Marital conflicts are also very prominent in later life. Though, it is really hard for single (widow, unmarried, and divorced) women to survive solely in this world. Everyone needs somebody to share last stage of life. To discuss daily life issues to spend time with someone as a partner. It has been observed that married elderly spend healthy life as compared to unmarried. Absence of partner at the last stage of life pushed an individual in to depression. Single/unmarried older adults are more vulnerable to depression as compared to married older adults [10, 20].

Similar to rest of South Asian countries, extensive family system has been practiced in Pakistan by many families [6]. It is assumed that extensive family system is a healthy family system. Number of people was available in joint family system, to play care taker role for their older adults. Love and respect are the main source of happiness for elderly in joint family system. They had been surrounded by their second or third generation and elderly smoothly passed their last stage of life [7]. But in present period several families in Pakistan start adopting nuclear family system because of inflation and growing trend of modernization. They badly indulge in race of mechanical world and forget about the well-being of their assists in the shape of elders of the family. A study conducted Karachi by in Pakistani researchers elaborated that nuclear family system significantly strong predictor of depression for older adults [6, 21].

Findings of the present study and Systematic literature review demonstrated that geriatrics who are financial strong, have source of earning, living in their own house, have no tension of billing, feed by significant others and on pension are less susceptible to depression as compare to elders who are un employed, have no source of income and not supported by the family. Study documented that awfully Pakistan ranked on 9th position in the race of low income holder countries with increasing number of population [22, 23].

## 5. Conclusion and Recommendation

It is concluded that elderly women undergo late life depression more than men. Widowhood, family conflicts, unsatisfactory family attitude, financial instability, communication gap, death of significant, unfulfilled early life desires, feeling of guilt, and later life general medical condition collectively act as strong predictorsof depression among older adults.

It is highly recommended to future researchers to design qualitative study along with quantitative study for in depth finds. Some questions of the interview would design open ended for comprehensive analysis.

In the beam of the findings of present study, it is immense need in Pakistan to design community support programs, welfare centers, policies for older adults' rights, grants or pensions from government sectors, and medical expense arrangements for older adults.

## Figures and Tables

**Table 1:** Frequency of various demographic variables.

Various demographic variables	*f*	%
Gender		
Male	158	51
Female	152	152
Marital status		
Married	201	65
Unmarried	109	35.16
Family system		
Nuclear family	190	61.2
Extensive family	120	38.71
Status of employment		
Employed	125	40.32
Unemployed	185	59.68
Source of income		
Self	123	40.32
Siblings/family	146	47.1
Pension	39	12.6
Living in own house?		
Yes	121	39
No	180	58.1
Having peer group		
Yes	220	70.1
No	90	20.03
Any physical aliment?		
Hypertension	52	16.77
Diabetes	67	21.61
Other body complaints	156	50.32
Not as such	37	11.93
Clinically diagnosed with mental ailment?		
Yes	42	13.55
No	268	86.45

**Table 2:** Mean differences in various demographic variables among elderly (*N* = 310).

Variables	M	SD	*t*	*P*	95% CI		Cohen's *d*
					LL	UL	
Gender							
Male (*n* = 158)	9.21	2.5	−2.69	0.008	−1.57	−2.44	0.3
Female (*n* = 152)	101.1	3.41					
Marital status							
Married (*n* = 201)	9.25	2.75	−3.24	0.001	−1.83	−0.45	0.4
Unmarried (*n* = 109)	10.4	3.31					
Family system							
Nuclear family (*n* = 190)	10.7	2.3	6.3	0.001	1.14	2.17	0.8
Extensive family (*n* = 120)	9.01	2.20p					
Status of employment							
Employed (*n* = 125)	8.6	1.8	−10.1	0.001	−2.90	−1.95	1.2
Unemployed (*n* = 185)	11	2.3					

*P < 0.05, df = 308.
